# Factors that influenced county system leaders to implement an evidence-based program: a baseline survey within a randomized controlled trial

**DOI:** 10.1186/1748-5908-5-72

**Published:** 2010-10-06

**Authors:** Wei Wang, Lisa Saldana, C Hendricks Brown, Patricia Chamberlain

**Affiliations:** 1 Dept. of Epidemiology and Biostatistics, College of Public Health, University of South Florida, 13201 Bruce B Downs Blvd., MDC 56, Tampa, FL 33612, USA; 2 Center for Research to Practice, 12 Shelton McMurphey Blvd., Eugene, OR 97401, USA; 3 Center for Family Studies, Department of Epidemiology and Public Health, University of Miami Miller School of Medicine, 1425 NW 10th Avenue, Miami, Florida 33136, USA

## Abstract

**Background:**

Despite the burgeoning number of well-validated interventions that have been shown in randomized trials to produce superior outcomes compared to usual services, it is estimated that only 10% of public systems deliver evidence-based mental health services. In California, for example, more than 15,000 children are placed in group homes or residential centers with some evidence of iatrogenic effects. The present study evaluates the willingness among county leaders of child public service systems to adopt a new evidence-based model, Multidimensional Treatment Foster Care, (MTFC), as a way to decrease the prevalence of out-of-home placements. Specifically, the study examines how county-level socio-demographic factors and child public service system leaders' perceptions of their county's organizational climate influence their decision of whether or not to consider adopting MTFC.

**Methods:**

Two levels were examined in this study: Stable and historical factors from 40 California counties gathered from public records including population size, number of entries into out-of-home care, financing of mental health services, and percent minority population; and system leaders' perceptions of their county's organizational climate and readiness for change measured via a web-based survey. The number of days-to-consent was the primary outcome variable defined as the duration of time between being notified of the opportunity to implement MTFC and the actual signing of a consent form indicating interest in considering implementation. Survival analysis methods were used to assess the predictors of this time-to-event measure. The present study is part of a larger randomized trial comparing two methods of implementation where counties are randomized to one of three time cohorts and two implementation conditions.

**Results:**

The number of entries into care was the primary predictor of days-to-consent. This variable was significantly correlated to county size. System leader's perceptions of positive climate and organizational readiness for change also contributed to but did not mediate or moderate the days-to-consent.

**Conclusions:**

System leaders' decision to consider implementing a new evidence-based model was influenced most by their objective need for the program and next by their perception of the county's organizational climate and motivation to change. These findings highlight the importance of understanding the fit between the needs of the systems or agencies and the potential for addressing those needs with the proposed new program.

## Background

Despite the increased calls for an emphasis on outcome accountability, performance-based contracting, and the adoption of evidence-based programs (EBPs) into routine practice settings, little is known about what influences decision makers in public systems to approve or disapprove of the implementation of such evidence-based practices in their individual service settings. A dearth of knowledge exists regarding how local contextual factors or organizational characteristics are related to the decision to implement evidence-based practices [1]. This is not surprising given that the majority of empirical studies on implementation have focused solely on a limited number of factors that mostly examine the performance and reactions of front line providers [2]. The scarcity of studies on the early stages of implementation and our limited knowledge about the decision-making process of system leaders results in an insufficient amount of empirical data to guide the strategic development of new EBPs or services aimed at increasing the rate of adoption. This lack of knowledge is a problem identified in the National Institutes of Health Roadmap Initiative, the National Institutes of Mental Health (NIMH) report Bridging Science and Service, and the National Academy of Sciences Institute of Medicine report on prevention of mental disorders and drug abuse [3].

The current analysis aims to fill a portion of this knowledge gap by identifying factors that significantly influenced system leaders' decisions to implement an EBP in the context of a statewide implementation trial in 40 California counties. Specifically, the study centered on the adoption of a single EBP; Multidimensional Treatment Foster Care (MTFC). None of the 40 counties had used MTFC at the onset of the study, which is a large randomized trial testing two methods of implementation for scaling-up MTFC in non-early-adopting California counties. System leaders (identified as directors of the three key child service systems: child welfare, juvenile justice, and mental health) in each county all received a standard invitation to consent to participate. Signing the consent to participate did not commit the county to implement MTFC, but rather to seriously consider implementation. No county could participate in the study without the consent of at least one of the three directors.

MTFC is an EBP intended to reduce placements in group/residential care for children and youth with severe behavioral and mental health problems. MTFC was named in 2009 as a top-tier practice by the Coalition for Evidence Based Policy and has been rigorously evaluated in a number of randomized trials [4-6]. MTFC is implemented in locally recruited foster homes by a team that includes a supervisor, two therapists, and other part-time staff (*e.g*., foster parent recruiter/trainer, skills coaches for youth). An MTFC team serves 10 to 12 youth for an average length of stay of six to nine months. The counties included in this study were non-early adopters in that they had not responded to previous opportunities or engaged in efforts to bring MTFC into their communities. Participation in the study offered all counties the opportunity to have a team of staff trained in the MTFC program (with all travel and consultation costs during implementation for MTFC practitioners included). Counties paid for all continuing services costs.

The current analysis examined two sets of factors thought to influence the directors' decision to consent to participate in the study. The first set included historical stable factors including county population characteristics. County population characteristics were defined at stable because they were not changeable due to study manipulation. The four stable factors examined were population size, percentage of minority residents, the previous annual number of children and youth placed in out-of-home care, and the previous annual dollars spent on publicly funded inpatient and outpatient mental health services. The second set of variables included system leaders' perceptions of organizational factors thought to influence implementation. Because these were potentially changeable as a function of participation in the study, they were defined as dynamic factors, and included system leaders' perceptions of their county's organizational climate, readiness, and motivation to change. Both the separate and interacting effects of stable and dynamic factors at baseline in relation to system leaders' decisions to consider implementing the MTFC EBP are examined. Of specific interest is whether the interplay of these factors affected the length of time it took system leaders to decide whether or not to sign the study's consent-to-participate form. Use of time-to-event data in implementation and dissemination studies has recently emerged as a relevant method to gauge meaningful progress (Trochin, unpublished presentation). *A priori*, it was anticipated that population size and need, defined by the number of youth who were being placed in out-of-home care, would strongly affect duration for consent to participate. In addition, the previous amount of dollars spent annually on mental health services was also expected to predict whether system leaders would consider implementing a new practice. It was hypothesized that the organizational climate and motivation for change in a county (*i.e*., dynamic factors) might interact with the socio-demographic characteristics of each county (*i.e*., stable factors). In particular, it was expected that quicker decisions to adopt would be observed from those counties where there was a high need and a climate conducive to change.

## Methods

### Procedures

Procedures for this study were part of a larger ongoing randomized implementation trial to scale up the MTFC model in the state of California under two implementation conditions (R01MH076158-01A1). In 2004, four years before this trial began the California Institute of Mental Health had extended a general invitation to all California counties to receive training in MTFC. At that time, nine of the 58 counties elected to participate; these early adopting counties were excluded from the current study. In addition, eight other counties were excluded that had a low 'need' for MTFC, defined as having fewer than six entries in to group care (*i.e*., the target population for the MTFC model) measured during two snapshot days in 2004. One additional county was excluded that was involved in a class action lawsuit that hindered their participation. The remaining 40 counties were targeted for recruitment into the study.

### Recruitment

Using a standard protocol, the directors of child welfare, mental health, and juvenile justice systems in each of the 40 counties were invited to participate. Introductory letters were sent to each of the system leaders describing the purpose of the study and stating that their county would have an opportunity to participate in a staged rollout of MTFC. The letter briefly described the evidence base for MTFC, as well as the staff training and ongoing consultation that counties would receive if they elected to participate. Further, the directors were informed that their county (along with 39 other counties) would be randomly assigned to participate in one of two methods of implementing MTFC: working in a Community Development Team (CDT) with up to six other counties, or working individually (IND) with trainers to implement the model. As reported in Chamberlain *et al*., the intervention condition assignments did not affect the system leaders' decision to participate [7]. System leaders also were informed that because of the large numbers of counties involved, implementation start dates would be staggered and counties would be randomly assigned to one of three timeframes (cohorts) for participation. Each cohort was spaced 12 months apart. Two weeks after sending the introductory letter, a second letter was sent with an appended consent form that informed counties of their intervention condition assignment and cohort. A study recruiter then contacted system leaders by telephone to address their questions and encourage them to consent to participate. If the leader had already chosen to sign the consent prior to being contacted by the recruiter, this time was used to answer any outstanding questions. The time between receiving the second letter to signing the consent form is used as the primary outcome variable for the current analyses.

### Data collection

To evaluate study hypotheses, both stable and dynamic factors were considered. Data on stable factors were gathered from public records, and data for dynamic factors were collected from all consenting county system leaders at baseline via a secure web-based survey. Thus, stable factor data were available for all participating counties, and dynamic data were available only for those counties with at least one consenting system leader.

### Participants

Of the 40 eligible counties, 37 had at least one system leader consent to participate. A total of 80 system leaders completed questionnaires and were included for analyses of dynamic factors. Of these 80 participants, the majority was female (67.5%) and Caucasian (87.5%; 2.5% Asian; 3.8% African-American; 2.5% Hispanic/Latino; 3.8% Other or mixed ethnic minority). The average age of participants was 51.9 years (SD = 7.40) and the average number of years post-graduation was 20.0 (SD = 10.98). Twelve counties had one respondent, 11 counties had two respondents, 10 counties had three respondents, and four counties had four respondents (average = 2.16 (SD = 1.01) respondents per county). Data from all responders were used to measure dynamic factors.

### Measures

#### Days-to-Consent

Days-to-consent was defined as the duration between the date that a county was first formally invited to participate (*i.e*., date they received the second letter) and the date that the first system leader signed a consent form. Although all three system leaders from each county were invited to participate (*i.e*., the directors of mental health, child welfare, and juvenile justice systems), the days-to-consent variable was calculated based on the first consent received. For analyses, this variable was treated two ways: First as a time-to-event outcome with right censoring for non-consenting counties (n = 3), and second as an ordinal scale by coding the duration into five categories with break points of 31, 90, 300, and 730 days. The five categories could be interpreted as: eager to participate (responded within one month); needed time to decide (responded between one month and three months); hesitant to participate (responded between three months and ten months); reluctant to participate (responded between ten months to two years); and non-responding. For those three counties that were treated as right censored, their time-to-consent was coded as non-responding (*i.e*., greater than two years, the maximum time available to consent for this study).

#### Stable factors

Data on stable factors were gathered from 2003 public statistics and included: county population, number of youth entries in to out-of-home care, Short-Doyle/medical penetration rate, and percent minority population. The Short-Doyle/medical penetration rate is the percentage of early periodic screening, diagnosis, and treatment (EPSDT) eligible Medicaid beneficiaries (assigned to a particular county) who are enrolled clients of that county's mental health plan. For example, a penetration rate of 5% means that 5% of eligible children were recipients of mental health services provided by the corresponding county during the specified fiscal year. These four factors were chosen because they were thought to be meaningful and relevant indicators that might influence the system leader's decision about whether or not to implement MTFC.

Descriptive analyses were conducted on the stable factors in order to transform variables and examine correlations prior to conducting survival analyses. Table 1 shows descriptive statistics of the stable factors across the 40 counties and their interrelationships. Population size and number of entries in to out-of-home care were highly correlated (r = 0.87, p < 0.01). The average number of youth entries in to out-of-home care was 1,396 (SD = 866) per 1,000,000 people per year, and in absolute numbers the average number of youth in out-of-home care per county was 515.7 (SD = 853.3). The average of Short-Doyle/medical penetration rate was 6.98% (SD = 2.26%). Minority percentage of the population ranged from 9.1% to 94.9% (median = 53.6%) with larger counties having a higher proportion of minority residents (r = 0.64; p < 0.01) but also lower per capita youth entries in to out-of home care (r = -0.32, p < 0.05) and lower penetration rates (r = -0.47, p < 0.01). Counties with a greater percentage of minority residents had lower penetration rates (r = -0.52, p < 0.05).

**Table 1:** Demographics of Stable Factors

								Spearman Correlation			
Stable Factors	Mean	St. d.	1^st ^Quartile	Median	3^rd ^Quartile	Skewness	Kurtosis	Entries	Per capita entries	Per capita financing	Percent minority
Population (in 1000)	407.6	486.8	55.4	203.8	476.1	1.58	1.51	0.87**	-0.32*	-0.47**	0.64**
Entries in to out-of-home care	515.7	853.3	79.3	246.5	445.5	3.1	10.8		0.12	-0.46**	0.64**
Per capita entries in to residential care (per 1000, 000 people)	1395.5	865.7	723.2	1190.2	1724.1	0.91	-0.04			0.11	-0.15
Short-Doyle/medical penetration rate (%)	6.98%	2.26%	5.26%	6.78%	8.23%	0.57	0.12				-0.52*
Percent minority population	64.6%^†^	21.8%	32.2%	53.6%	64.7%	-0.01	-0.89				

^† ^: Weighted average by population size.

**: p-value <0.01; *: p-value <0.05

Several alternative transformations of the data were considered. For example, counties were dichotomized into rural (population ≤ 200,000) or non-rural (population > 200,000) in some of the analyses. Further, logarithmic transformations of the variables 'population' and 'number of youth entries in to care' allowed for examination of linear combinations on the logarithmic scale of whether the magnitude (*i.e*., log entries) or proportion (*i.e*., log [entries/population] = log [entries] - log [population]) were more effective predictors by examination of the multiple regression coefficients.

#### Dynamic factors

Dynamic factors were hypothesized to mediate the degree to which MTFC would be successfully implemented in participating counties. Measures of dynamic factors were adaptations of two standardized organizational measures, the Organizational Readiness for Change (ORC) [8] and the Organizational Climate Survey (OCS) [9]. Adaptations to the measures included slight wording changes for language consistency across measures, and item deletion to avoid duplication of items that were the same across the two measures.

#### The ORC

The adapted ORC (system leader version) [8] included 66 items on a five-point Likert Scale that target constructs in a theoretical process model of program change [10]. Domains measure motivational readiness (*e.g*., perceived need and pressure for change, immediate training needs), adequacy of resources (*e.g*., offices, staffing, training, computer access, electronic communications), staff attributes (*e.g*., growth, efficacy, influence, adaptability), organizational climate (*e.g*., clarity of mission, cohesion, autonomy, communication, stress, change), and training exposure and utilization (*e.g*., frequency of attendance, adoption of new techniques). Each domain has demonstrated satisfactory reliability, validity, and internal consistency at the staff, director, and program levels of evaluation with scale reliabilities ranging from 0.44 to 0.80 [8,11].

#### Organizational climate

The OCS [9] was used to assess system leader perceptions of organizational climate. The Climate scale is rated on a five-point Likert scale (1 = not at all; 5 = to a very great extent) and yields higher order scales including structure (*e.g*., hierarchy of authority), work attitude (*e.g*., job satisfaction, organizational commitment), and climate (*e.g*., emotional exhaustion, fairness, personal accomplishment, role clarity) with alphas for all subscales ranging from 0.68 to 0.92. This scale has been linked to organizational outcomes in both the child welfare and juvenile justice systems.

#### Analytic strategy

In the current analyses, days-to-consent was treated as a time-to-event outcome. A nonparametric survival function of the days-to-consent was computed using the Kaplan-Meier estimator [12]. Estimates of covariate effects and hypothesis testing were calculated using Cox proportional hazards modeling [13] and the nonparametric logrank comparison test [14]. The 'hazard' under a Cox proportional hazard model was interpreted as the instantaneous probability of the county system leader signing a consent letter in the next small interval of time. The proportional hazards survival models also describe how the underlying hazard varies in response to explanatory covariates, including both stable and dynamic factors. When the hazard ratio (HR) is greater than one, consent occurs faster for larger values of the covariate compared to smaller values. When the HR ratio is less than one, consent occurs slower for larger values of the covariate. The proportional hazards assumption was examined through diagnostic plots [15], as well as chi-square tests of polytomous regression modeling formed by trichotomizing the time-to-event outcome.

When significant relationships were found between stable and dynamic factors on the outcome variable days-to-consent, further testing was conducted to examine the presence of mediation and moderation effects. Such information could provide useful insights to inform the design of future studies on implementation of EBPs. To test for a mediation impact, an additional linear regression model was run to obtain estimates of the regression coefficient that depicted the relationship from stable factors to dynamic factors. Next, this was paired with the estimated regression coefficients obtained in the proportional hazard model that depicted the relationship between dynamic factors and the days-to-consent outcome. Finally, the products of coefficients method was used to test the significance of the mediation effect [16-18].

To test for moderation effects, the same proportional hazard modeling approach was used and interaction terms between stable factors and dynamic factors were added. Significant interaction terms indicate moderation effects of dynamic factors.

## Results

### Days-to-consent descriptive outcomes

Days-to-consent ranged from four to 533 days, with 19 counties (47.5%) consenting before 31 days, 10 counties (25.0%) consenting between 32 days and 90 days, three counties (7.5%) consenting between 91 days and 300 days, and five counties (12.5%) consenting between 301 days and 533 days. Three counties (7.5%) had yet to consent by two years.

### Influence of stable factors on days-to-consent

**Figure 1:**
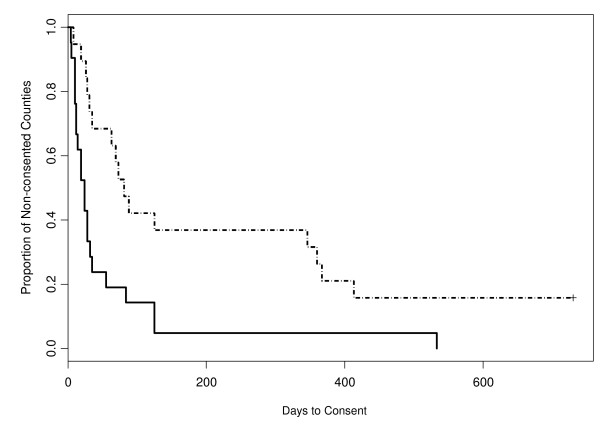
**Survival curves for days-to-consent for rural and non-rural counties**. *The two curves depicted Kaplan-Meier estimator for days-to-consent outcome of rural (population ≤ 200,000) and non-rural counties (population > 200,000) from day 0 to day 733, where rural counties is shown in dotted and non-rural counties in solid lines.

The first hypothesis was that stable factors, such as population size, would significantly predict a county's willingness to consider implementing the MTFC model. As shown in Figure 1, the days-to-consent curves depict the change in consent rates across time, with the solid line representing the non-rural counties and the dashed line representing the rural counties. Results of this study show that large counties consented sooner than small counties. For example, at day 200, fewer than 10% of the non-rural counties had yet to consent; although this proportion was close to 40% for the rural counties at the same time point. Similarly, using a logrank test, rural counties took significantly longer to consent than non-rural counties (p = 0.003). Proportional Hazard modeling further revealed that the hazard ratio, 2.64, between rural counties and non-rural counties favored non-rural counties consenting much faster (95% CI = 1.35, 5.18).

**Figure 2:**
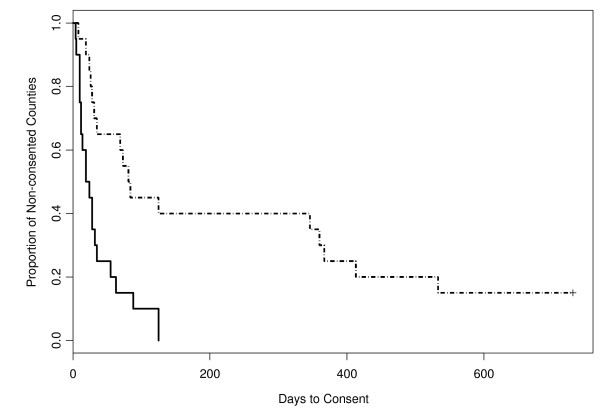
**Survival curves for days-to-consent for high entries and low entries counties**. *The two curves depicted Kaplan-Meier estimator for days-to-consent outcome of counties with low number of youth entries (number of entries ≤ 246.5) and high number of entries (number of entries > 246.5) from day 0 to day 733, where low entry counties is shown in dotted and high entry counties in solid lines.

The second hypothesis considered the influence of county need on system leaders' days-to-consent. The number of annual entries of youth in to care was viewed as an indicator of the need for a county to consider implementing MTFC. As shown in Figure 2, the days-to-consent curves depict the change in consent rates across time, with the solid line representing the counties with high number of entries (number of entries > 246.5, the median of all 40 counties in the study) and the dashed line representing the remaining counties with less than the median entries. Counties with high numbers of entries consented significantly faster than those with low numbers of entries (p < 0.01, logrank test). Proportional hazard modeling further revealed that the hazard ratio between low-entry counties and high-entry counties was 3.44 (CI = 1.65, 7.17). In other words, at a given time point, the instantaneous chance of consent from high-entry counties was nearly three and one-half times more than the chance of consent from low-entry counties.

Neither the Short-Doyle penetration rate nor the percent minority population reached individual standard significance levels as predictors for the days-to-consent (p = 0.08 for penetration rate and p = 0.06 for percent minority). In the next step, however, all stable factors were included in the same model to assess their combined influence.

### Combined influence of stable factors

The first time-to-event multiple predictor model included all four baseline stable factors (county population, percentage of minority residents, previous annual number of youth placed in out-of-home care, and previous annual dollars spent on outpatient mental health services). Similar to their individual influence, penetration rate and percent minority population were not statistically significant (p = 0.60 for penetration rate and p = 0.72 for percent minority). Thus, these two predictors were excluded in subsequent regression analysis.

Both the log of population size (HR = 1.77, 95% CI = (1.30, 2.41), p < 0.001) and log per capita entries (HR = 1.90, 95% CI = (1.11, 3.26), p = 0.02), were significant predictors of days-to-consent, with larger population size and higher per capita entries associated with a higher likelihood of early consent. Estimates of the regression weights on population size and per capita entries had nearly the same values, suggesting that the linear combination log (Entries) (= log [Population] + log [Entries/Population]) might have been a sole significant predictor. This was verified when a second model was fit with (log) number entries and (log) population size as the predictors; population size was no longer significant (p = 0.95) while number of entries remained significant (HR = 1.77, 95% CI = (1.04, 3.00), p < 0.04). This verified that the number of entries in to out-of-home placements was the dominant predictor of days-to-consent and was more important than county population size.

### Influence of dynamic factors

As a continuation of the proportional hazards model that was used to evaluate the impact of stable factors to days-to-consent, dynamic factors were added to the model to further examine the hypothesis that baseline dynamic factors also influence a system leader's rate of consent. The model included all 37 counties who had at least one system leader complete a baseline survey that included the ORC and the OCS measures. The dynamic factor measures were averaged over responses from all system leaders in a county. The predictability of the dynamic factors first was examined individually, controlling for number of youth entries in to care. Among the five subscales in the ORC scale, adequacy of resources (p = 0.58), staff attributes (p = 0.27), organizational climate (p = 0.57), and training exposure and utilization (p = 0.78) were not significant, but motivational readiness (p = 0.002) was. None of the subscales in the OCS were significant; structure (p = 0.17), attitude (p = 0.93), and climate (p = 0.10).

Next, the combined predictability of these dynamic factors was examined. Both significant and non-significant factors discovered in the previous step were included in the model to avoid omitting significant predictors. That is, some significant factors might have only been detected in the combined model due to the small standard deviation for the error terms. A backward stepwise selection procedure was then used to select only significant predictors to be included in the proportional hazard model. Number of youth entries in to out-of-home care was included in the model during the selection process. The result showed that in the combined final model, the climate subscale from the OCS (HR = 1.22, 95% CI = (1.04, 1.42), p = 0.01) and motivational readiness from the ORC (HR = 1.25, 95% CI = (1.10, 1.43), p = 0.001) were positively related to faster consent, and number of youth entries in to care (HR = 1.58, 95% CI (1.18, 2.12), p = 0.002) remained significant (see Table 2).

**Table 2:** Proportional Hazard Model Fitting Result for Baseline Stable Factors and Dynamic Factors (n = 36)

Covariate	Est.	HR*	95% CI of HR	S.E.	Est./S.E.	p-value
Log(Entries)	0.46	1.58	(1.18, 2.12)	0.15	3.05	0.002
OCS - Climate	0.20	1.22	(1.04, 1.42)	0.08	2.51	0.012
ORS - Motivation	0.23	1.25	(1.10, 1.43)	0.07	3.48	0.001

* Hazard Ratio = exp (Est.)

### Mediation and moderation effects

Given that population size was not significant when county need was entered into the model, a meditational analysis was conducted to examine if the two significant dynamic factors, the climate subscale from the OCS and the motivational readiness subscale from the ORC, mediated the relationship between county need (*i.e*., number of youth entries in to care and days-to-consent). Using the standard products of coefficients estimate and standard error for mediation [16,17], the climate scale from the OCS was determined not to be a significant mediator of entries (p = 0.98). The same held true for the motivational readiness subscale from the ORC (p = 0.67). Thus, there was no significant mediation effect at baseline, and these two factors were independent predictors of consent.

Next, potential moderation effects of dynamic factors were tested by examining their interaction with the number of entries into care (*i.e*., county need). None of the interactions were significant (p-values range = 0.23 to 0.80); both the climate in the OCS and motivational readiness in the ORC scales contributed in an independent fashion to the outcome of days-to-consent.

## Discussion

The present study is the first to our knowledge to examine predictors of system leaders' decisions to consider implementation of an EBP where the leaders were non-early adopters. All of the participating California counties had declined prior opportunities to implement the practice. Given that up to 90% of public systems do not volunteer to adopt EBPs, more information about the best ways to garner interest among non-early adopters is needed to facilitate increased penetration of EBPs into the majority of routine care settings. Findings from this study suggest that the county system leaders based their decisions about considering implementation on their own objective need. The MTFC model was designed to prevent placements in to high-end out-of-home care for children and teenagers with severe mental health, drug use, and emotional problems. The primary finding from this study was that the number of entries in to out-of home care was the strongest predictor of rate of consent by county system leaders. As may be expected, system leaders in larger counties made the decision to participate in the study more quickly than those in smaller rural counties who presumably had less to benefit because of the limited number of youth affected in their counties. Although the costs for training, travel, and consultation to implement were supported by the grant, the costs associated with changing current practices (*e.g*., overcoming inertia) when implementing a new intervention make it less economical for counties serving few families [19]. In addition, smaller counties generally have fewer resources to implement a new model, especially one that is as staff intensive as MTFC. The prior amount of funds spent on mental health services was not predictive of days-to-consent nor was the proportion of minority population in the counties.

Additionally, positive scores from system leaders on organizational climate and motivation for change predicted shorter response times to consent. However, neither positive organizational climate nor motivation to change scores mediated the number of entries in to care at baseline, indicating that these factors were independent predictors of time-to-consent once need was accounted for. Similarly, there was no moderation effect found for climate or motivation measures.

While this is possibly the largest study of county-level predictors about factors that affect implementation of an EBP, more complex interrelationships than those examined here cannot be ruled out. It is well known that the statistical power for tests of interactions and mediation are smaller than those for a comparable sized main effect [17,20]. Thus, the fact that no significant moderators or mediations were found may be due to limited power with data from 37 counties. Although big enough to detect a large effect, in order to detect either medium or small effects, a much larger sample size would be required. Because the test for mediation requires that both the path from stable factors to dynamic factors, and from dynamic factors to days-to-consent are significant, if either or both detect only a medium or small effect, the sample size from this study would be insufficient [18]. Another major limitation of the study is that other unmeasured factors might be key predictors in the decisions that system leaders make about whether or not to implement a given practice at a given time.

The time-to-event modeling method that was used to examine predictors of duration to a critical first stage in the implementation process may also be appropriate for other implementation evaluations. This approach could be successfully employed for all later implementation stages. As long as there is a standardized invitation to start time and a measurable behavior such as consent, hiring staff, completion of staff training, or start date for new services, the time-to-event for completion can be readily measured. Indeed, our randomized trial evaluation plan relies heavily on the use of a Cox proportional hazards model to examine the time until completion of multiple stages of implementation using the stages of implementation completion measure developed for this trial [21]. In addition to modeling the time to complete multiple stages, further modeling also can examine quality (fidelity) and quantity (amount of services provided) associated with the completion of a stage. These two dimensions were not relevant in our current study but will be in future research. Understanding the effects of an implementation or system level translational strategy [22] will require attention to the quantitative and qualitative output dimensions and their sustainability or longevity as well as the speed at which they are implemented.

The study's primary finding was that directors of the three key child service systems were most influenced by their objective need for the intervention being offered to attempt to solve a real-world problem (*i.e*., placing children in out-of home care). This speaks to the importance of a program's fit into the local needs and priorities of individual counties. Careful preparation and partnering with system leaders is needed to analyze local needs and existing service use patterns. The speed at which system leaders decided to participate may not be an accurate predictor of the county's eventual success in implementation or in the county's subsequent sustainability of MTFC programs. These questions will be addressed as the study progresses over time.

## Competing interests

PC is a partner in Treatment Foster Care Consultants Inc, a company that provides consultation to systems and agencies wishing to implement MTFC.

## Authors' contributions

WW led the analyses of the data in this manuscript, contributed substantially to the conceptualization of the study hypotheses, interpretation of the data, and writing of the manuscript. LS contributed to the conceptualization of the study hypotheses, specification of the study methods and procedures, and contributed substantially to the writing of the manuscript. CHB contributed to the conceptualization of the larger randomized trial on which the study data are based, and contributed to the conceptualization of the hypotheses, data analysis, and writing of the manuscript. PC is the P.I. on the randomized trial that forms the basis for this study, oversaw all data collection, and contributed to the conceptualization, design, and writing of the manuscript.
